# Seizures, climate and pollution: is there evidence of an association?

**DOI:** 10.3389/fpubh.2025.1708538

**Published:** 2025-11-13

**Authors:** Cecilia Llobet, Montserrat Martinez-Alonso, Elena Justribó, Jaume Ortet, Oriol Yuguero

**Affiliations:** 1 ERLab, Research on Emergencies, Biomedical Research Institute of Lleida (IRBLLEIDA), Lleida, Spain; 2 Systems Biology and Statistical Methods for Biomedical Research Group, Institute for Biomedical Research Dr. Pifarré Foundation, IRBLLEIDA, Lleida, Spain; 3 Faculty of Medicine, University of Lleida, Lleida, Spain

**Keywords:** epilepsy, seizures, air pollution, nitrogen dioxide, temperature, environmental health

## Abstract

**Background:**

Epilepsy, a chronic neurological disorder affecting more than 65 million people worldwide, is characterized by recurrent seizures caused by abnormal neuronal discharges. Beyond its diverse etiologies, growing evidence suggests that environmental and meteorological factors, including air pollution, may influence seizure occurrence.

**Objective:**

To evaluate the association between meteorological variables, air pollutants, and hospital admissions for seizures in the province of Lleida (Spain) over a 10-year period (2010–2019).

**Methods:**

Daily hospital admissions for seizures (ICD-10 code G40) were analyzed together with meteorological variables (temperature, atmospheric pressure, humidity, precipitation, and solar irradiation) and air pollutants (NO_2_, PM_10_, CO, O_3_, and SO_2_). Distributed lag non-linear models (DLNM) with quasi-Poisson regression were applied to estimate exposure–response relationships and delayed effects, adjusting for long-term trends, seasonality, and day of the week.

**Results:**

A total of 4,755 seizure-related admissions were recorded, representing 0.52% of all emergency visits. The frequency of seizures increased during colder months and periods of poor air quality. Low daily mean temperatures (<2.5th percentile) and high NO_2_ concentrations (>99th percentile) were both significantly associated with higher seizure risk (up to +40% and +42% relative risk, respectively; *p* < 0.05). The association with NO_2_ remained significant after adjusting for temperature.

**Conclusion:**

Cold temperatures and elevated NO_2_ levels are independently associated with an increased number of seizure-related emergency admissions. These findings highlight the potential neurological impact of air pollution and extreme weather, emphasizing the need for preventive public health measures and further research to clarify the underlying mechanisms.

## Background

Although the prevalence of epilepsy may appear moderate, its impact on patients’ quality of life is substantial, affecting education, employment, social relationships, and mental well-being (1). Epilepsy is a chronic, severe, and frequent neurological disorder that affects approximately 65 million people worldwide (2). It encompasses a group of diseases characterized by abnormal electrical discharges in the brain, resulting in sudden and recurrent seizures that may involve loss of consciousness, motor convulsions, and other neurological symptoms. These episodes can lead to significant morbidity, injuries, and even mortality, severely compromising quality of life (3).

The etiology of epilepsy is highly heterogeneous, including causes such as stroke, traumatic brain injury, tumors, genetic alterations, infections, metabolic disturbances, and autoimmune or immune-mediated conditions. However, numerous intrinsic and extrinsic factors may precipitate seizures in predisposed individuals. Among these, alcohol consumption, illicit drug use, sleep deprivation, stress, and environmental or meteorological changes have been identified as relevant triggers (4). In Spain, approximately 500,000 people live with epilepsy, and nearly 20,000 new cases are diagnosed each year, mainly among children and adults over 65 years old (5). Seizures account for between 1 and 3% of all emergency department (ED) admissions and up to 20% of urgent neurological consultations according to the Spanish Society of Neurology (SEN) (6). Clinicians frequently report temporal increases in seizure-related admissions, which some patients associate with seasonal or climatic variations.

The onset of seizures is determined by the interplay between intrinsic factors—such as the etiology of epilepsy, genetic background, and physiological state—and extrinsic factors, including medications, sleep patterns, and environmental exposures (7). Weather conditions exert a measurable influence on multiple physiological systems, and a growing body of evidence suggests that meteorological factors may modulate seizure occurrence (8). Previous studies have linked variations in atmospheric temperature, barometric pressure, humidity, and sunlight exposure to fluctuations in seizure frequency (9). Previous studies have linked epilepsy to climatic factors such as atmospheric temperature, atmospheric pressure, precipitation, and exposure to sunlight (10).

At the biological level, several mechanisms have been proposed to explain how external triggers might influence neuronal excitability. Psychoactive substance use can lower the seizure threshold by disrupting neurotransmitter balance (11). Stress and sleep deprivation induce neuroinflammatory responses and hormonal changes, such as elevated cortisol levels, that enhance neuronal hyperexcitability (12). Furthermore, transient receptor potential (TRP) ion channels expressed in the brain are sensitive to thermal variations and may directly modulate seizure susceptibility through temperature-dependent changes in neuronal firing (13). Poor housing conditions or inadequate insulation may expose individuals to temperature extremes or indoor pollution, indirectly influencing seizure occurrence (14).

In certain geographical areas, where there are significant variations in temperature and humidity throughout the year, these can directly influence the health of the inhabitants, especially those who are vulnerable to specific environmental factors, such as people with epilepsy or those who have experienced seizures (15).

Recent research has expanded the focus from meteorological conditions to air pollution, particularly fine particulate matter (PM_2.5_ and PM_10_). Epidemiological evidence consistently links PM_2.5_ exposure to respiratory, cardiovascular, and neurological diseases (16). Fine particles can penetrate deep into the respiratory system, cross biological barriers, and induce oxidative stress, inflammation, and immune dysregulation, promoting the release of cytokines such as IL-4, IL-6, IL-13, TNF-*α*, IL-17, and IFN-*γ* (17). PM_2.5_-bound chemical components exhibit enhanced toxicity and may disrupt gene expression through non-coding RNA mechanisms, amplifying neuroinflammatory and neurotoxic effects (17). However, most previous studies have focused on identifying pollution sources rather than characterizing direct health consequences, and the neurological impact of air pollution—particularly regarding seizure occurrence—remains underexplored.

Importantly, ambient particulate matter rarely exists in isolation. It commonly interacts with other pollutants such as nitrogen dioxide (NO_2_) and ozone (O_3_), which may potentiate each other’s effects on the respiratory, cardiovascular, and nervous systems. Understanding these synergistic interactions is essential for evaluating the cumulative burden of environmental exposures on neurological health.

In regions characterized by pronounced climatic variability, such as the province of Lleida in northeastern Spain, the combined influence of meteorological and air pollution factors on health outcomes may be especially relevant. Therefore, the objective of this study was to analyze the association between weather conditions, air pollutants, and seizure-related emergency department admissions over a 10-year period (2010–2019) in a population exposed to diverse environmental conditions.

## Methods

### Setting

Data on hospital admissions, meteorological variables, and air pollutants for the Spanish province of Lleida were collected for the period 2010–2019.

Lleida is a province with a population of approximately 0.44 million. Its capital, also named Lleida, accounts for about 32% of the provincial population, while the surrounding health region represents nearly 83% of the total population. According to the Köppen Climate Classification, the southern part of the province (including the capital) has a cold semi-arid climate (BSk), whereas the northern mountainous areas are characterized by temperate climates without dry seasons (Cfa–Cfb) and colder, wetter conditions in the Pyrenean region (Dfb–Dfc).

### Data

Daily hospital admissions for seizures were obtained from the Conjunto Mínimo Básico de Datos (CMBD), the mandatory national hospital discharge registry maintained by the Spanish Ministry of Health. The CMBD includes data from all public hospitals since 1997 and from private hospitals since 2016.

Cases were identified using the ICD-10-CM codes G40 (epilepsy and recurrent seizures), which encompass the majority of epileptic and seizure-related hospitalizations. Admissions coded as R56 (febrile convulsions) were excluded to minimize potential misclassification of acute, non-epileptic events. Nevertheless, the use of administrative data may still include a small proportion of cases related to non-epileptic events such as syncope or other causes of transient loss of consciousness (LOC).

The dataset did not include detailed clinical information, such as etiology or prior epilepsy diagnosis, and thus it was not possible to distinguish between acute symptomatic and unprovoked seizures. Data were accessed for research purposes between 15 July 2022 and 25 September 2022.

### Meteorological and air pollution variables

Meteorological data included daily (24-h) mean, minimum, and maximum values for temperature, relative humidity, and atmospheric pressure, as well as daily accumulated precipitation, global solar irradiation, and mean and maximum wind speed (measured at 2 meters height). These data were obtained from the Meteorological Service of Catalonia, using four automatic weather stations located in the province. The mean of these stations was computed as representative of the population-weighted average for the region, given the concentration of inhabitants in the capital area. We used air pollution data from a single monitoring station, which is the only station in the province that measures NO and NO_2_. In contrast, meteorological data were obtained from four different weather stations distributed across the region. That data was obtained in January 2023. Air pollution data were obtained from the Catalonian Air Pollution Monitoring and Forecasting Network (XVPCA). Measurements included daily means and maxima for NO_x_, NO, NO_2_, SO_2_, PM_10_, and O_3_, as well as the 8-h moving average for CO and O_3_. Because only one monitoring station in the province measures nitrogen oxides (NO and NO_2_), these data were used as reference for the entire study area.

European Air Quality Index thresholds were used to classify poor air quality: CO (10 mg/m^3^/h), NO_2_ (120 μg/m^3^/h), O_3_ (130 μg/m^3^/h), PM_10_ (24-h mean of 50 μg/m^3^), and SO_2_ (350 μg/m^3^/h).

### Statistical analysis

We summarized all the exposure and response variables with mean, standard deviation, coefficient of variation, median, interquartile range (IQR), quartile coefficient of dispersion (IQR divided by the sum of quartile 1st and 3rd) and maximum. For temperature measures, the coefficient of variation as well as the quartile coefficient of dispersion were computed in Kelvin units (1 K = 1C° + 273.15). For the pollution variables, the number of days with poor air quality due to each pollutant was also reported.

We used distributed lag non-linear models (DLNM) to estimate the relationship between hospital admissions caused by seizures and the exposure variables of weather and pollution in order to capture possible delayed effects. We fitted a time-series Quasi-Poisson regression model in which the exposure-response association was modeled using a quadratic B-spline with 3 internal knots placed at the 10th, 50th and 90th percentiles of location-specific exposure distribution. Each model took into account seasonality by including a natural cubic spline of the day of the year with 4 degrees of freedom and an interaction between this spline function and the indicator of year to relax the assumption of a constant seasonal trend. Long-term trends were controlled by including a linear term for the day of the study period. In addition, we included indicator variables for the day of the week and holidays. The lag-response association was modeled by a natural cubic spline with an intercept and three internal knots placed at equally spaced values in the log scale. To explore possible short as well as long delays in the effects of exposure variables, the lag period was tested at 3, 7 and extended up to 21 days to capture long lags related to weather or pollution variables. Moreover, we report the estimated relative effect by using the lag providing the minimum AIC.

The results were aggregated over all lags to obtain the overall exposure-response association curves. And were reported as relative risks in reference to the percentile of minimum hospitalizations (minY) estimated from each curve. This minY was replaced by the median or 50th percentile for minY values below 20th or above 80th percentile. These estimated exposure-response curves were used to quantify the percent relative effect of very extreme exposure values, defined as percentiles 1, 2.5, 97.5 and 99.

In case of significant associations with both weather and pollution variables, we used DLNM to assess if, once adjusted by the weather variable inside the model through natural splines with 4 degrees of freedom, the exposure to the pollutant keeps its significant association with hospital emergency room admissions. Although the models accounted for temporal confounders such as seasonality, long-term trends, day of the week, and public holidays, individual-level confounders (e.g., medication use, comorbidities, socioeconomic factors, or lifestyle habits) could not be included in the analysis because such information was not available in the CMBD dataset. Nevertheless, the time-series design inherently controls for stable population characteristics and slowly varying individual factors, reducing the likelihood of major confounding bias.

All analyses were performed with R software (18). The package DLNM was used (19). As stated by Gaspirrini, DLNMs is a “modelling framework to describe simultaneously non-linear and delayed effects of predictors and an outcome, a dependency defined as exposure-lag-response association.” In comparison with traditional time-series regression, DLNMs captures delayed (lagged) possible effects from environmental exposures more flexibly, allowing for non-linear relationships in both dimensions (exposure-response and lag-response). Since environmental exposures (air pollutants and weather) can show non-linear (J or U-shaped) possible effects on health, DLNMs is the best applicable methodology to answer to ours aims with a data-driven model, without having assumed any form of relationship.

### Ethics

The project was approved by the Research Committee of Lleida with ID 2323. All the research was conducted following Helsinki Declaration. All data was handled according to current European legislation. The researcher accessed it for research purposes on the 13th of September, 2022. The authors had no access to information that could identify individual participants during or after data collection. The research committee waived the requirement for informed consent.

## Results

**Figure 1:**
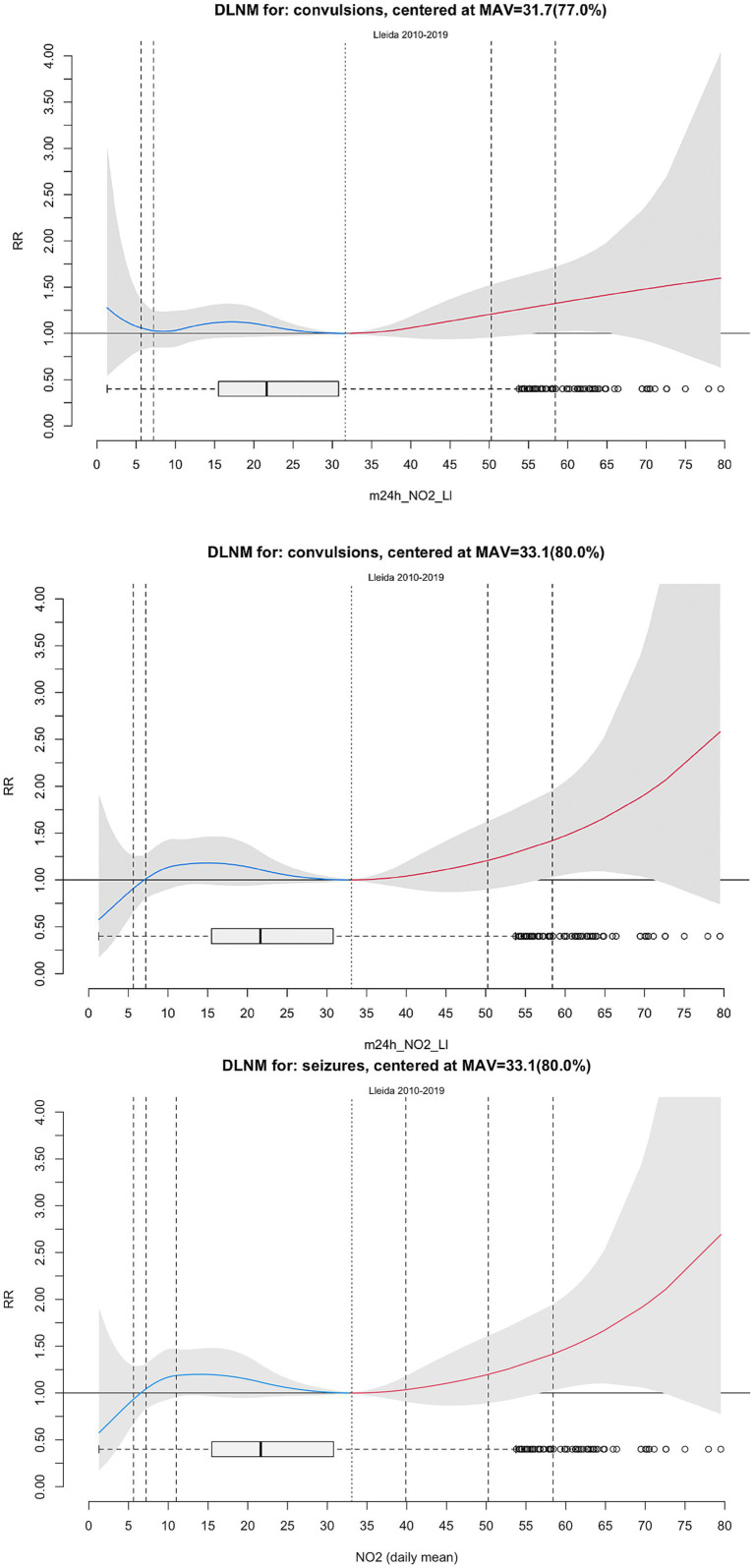
Distribution of hospital emergency room admissions for seizures per month, 2010–2019.

A total of 4,755 hospital emergency admissions for seizures were recorded from 2010 to 2019, representing 0.52% of all 908,003 emergency visits. Annual counts ranged from 117 to 667. The daily number of seizure-related admissions had a mean of 1.3 (median 1; range 0–11), while the monthly cumulative count had a mean of 39.6 (median 40; range 0–78). The highest median monthly admissions occurred in January (Figure 1).

Among meteorological variables, daily cumulative precipitation was the most variable during the study period (coefficient of variation = 3.9; mean = 1.0 mm; median = 0 mm) (Table 1). In contrast, the 24 h mean temperature showed low variability (CV = 0.03; mean = 14 °C).

**Table 1:** Descriptive summary.

	Mean	SD	St. mean	CV	Median	IQR	QCV	Max	nDgtL
Seizures	1.3	1.34	0.97	1.03	1	2	1	11	
Temperature (mean)	14.4	7.58	37.94	0.03	14.3	12.6	0.02	29.8	
Temperature (min)	8.34	6.95	40.51	0.02	8.2	11.3	0.02	22.5	
Temperature (max)	21.4	8.73	33.72	0.03	21.45	14.2	0.02	41.9	
Relative humidity (mean)	68.7	13.4	5.12	0.2	67.5	19	0.14	100	
Relative humidity (min)	42.5	18	2.36	0.42	38	22.5	0.28	100	
Relative humidity (max)	90.4	7.3	12.37	0.08	92.5	9	0.05	100	
Pressure (mean)	983	6.76	145.44	0.01	983.3	7.5	0	1.007	
Pressure (min)	981	7.21	136.09	0.01	980.8	7.7	0	1.005	
Pressure (max)	986	6.41	153.74	0.01	985.6	7.2	0	1.009	
Precipitation (24 h)	0.97	3.83	0.25	3.94	0	0.1	1	61.8	
Solar irradiation (24 h)	17.1	8.68	1.96	0.51	17.17	15.3	0.45	32.8	
Wind velocity (mean)	1.01	0.68	1.48	0.67	0.8	0.8	0.44	5.5	
Wind velocity (max)	5.7	2.6	2.19	0.46	5.1	3	0.28	18.5	
CO (8 h ma)	0.31	0.16	1.98	0.5	0.25	0.14	0.24	1.45	
CO (max)	0.43	0.29	1.49	0.67	0.3	0.2	0.25	3.5	0
NO_X_ (mean)	37.4	28.7	1.30	0.77	27.91	27.6	0.43	228	
NO_X_ (max)	94.6	85.9	1.10	0.91	66	72	0.47	807	
O_3_ (8 h ma)	69.5	31	2.23	0.45	73.56	44.6	0.31	141	
O_3_ (max)	80.3	31.4	2.55	0.39	83	41.8	0.25	164	143
PM_10_ (mean)	24.4	12.7	1.92	0.52	22.22	15.7	0.34	100	158
SO_2_ (mean)	2.17	1.36	1.59	0.63	1.91	1.46	0.38	18.9	
SO_2_ (max)	3.52	2.83	1.24	0.8	3	2	0.33	37	0
NO (mean)	11	14	0.79	1.27	5.45	11	0.67	109	
NO (max)	37.3	49.1	0.75	1.32	18	38	0.7	539	
NO_2_ (mean)	23.9	11.6	2.06	0.48	21.62	15.3	0.33	79.5	
NO_2_ (max)	50.8	27.5	1.84	0.54	44	32	0.33	227	76

SD, standard deviation; CV, coefficient of variation; IQR, interquartile range; QCD, quartile coefficient of dispersion (IQR divided by the sum of quartile 1st and 3rd); nDgtL, number of days with poor air quality due to pollutant maximum daily values exceeding the European limits.

Among pollutants, the most variable was the 24 h maximum concentration of nitrogen monoxide (NO) (CV = 1.3), more than twice the variability observed for the 24 h maximum of nitrogen dioxide (NO_2_), daily mean PM_10_, or the maximum 8 h moving average of ozone (O_3_), which had CVs of 0.54, 0.52, and 0.45, respectively. The mean concentrations of these pollutants were 37 μg/m^3^ for NO, 51 μg/m^3^ for NO_2_, 24 μg/m^3^ for PM_10_, and 70 μg/m^3^ for O_3_.

The concentrations of CO and SO_2_ did not exceed poor-air-quality thresholds on any day during the study period, whereas NO_2_, O_3_, and PM_10_ did. Poor air quality was recorded on 326 days (8.9%) over the 10 years. Specifically, the PM_10_ limit was exceeded on 158 days, O_3_ on 143 days, and NO_2_ on 76 days; 43 days showed simultaneous exceedance of PM_10_ and NO_2_, and 8 days for PM_10_ and O_3_.

**Figure 2:**
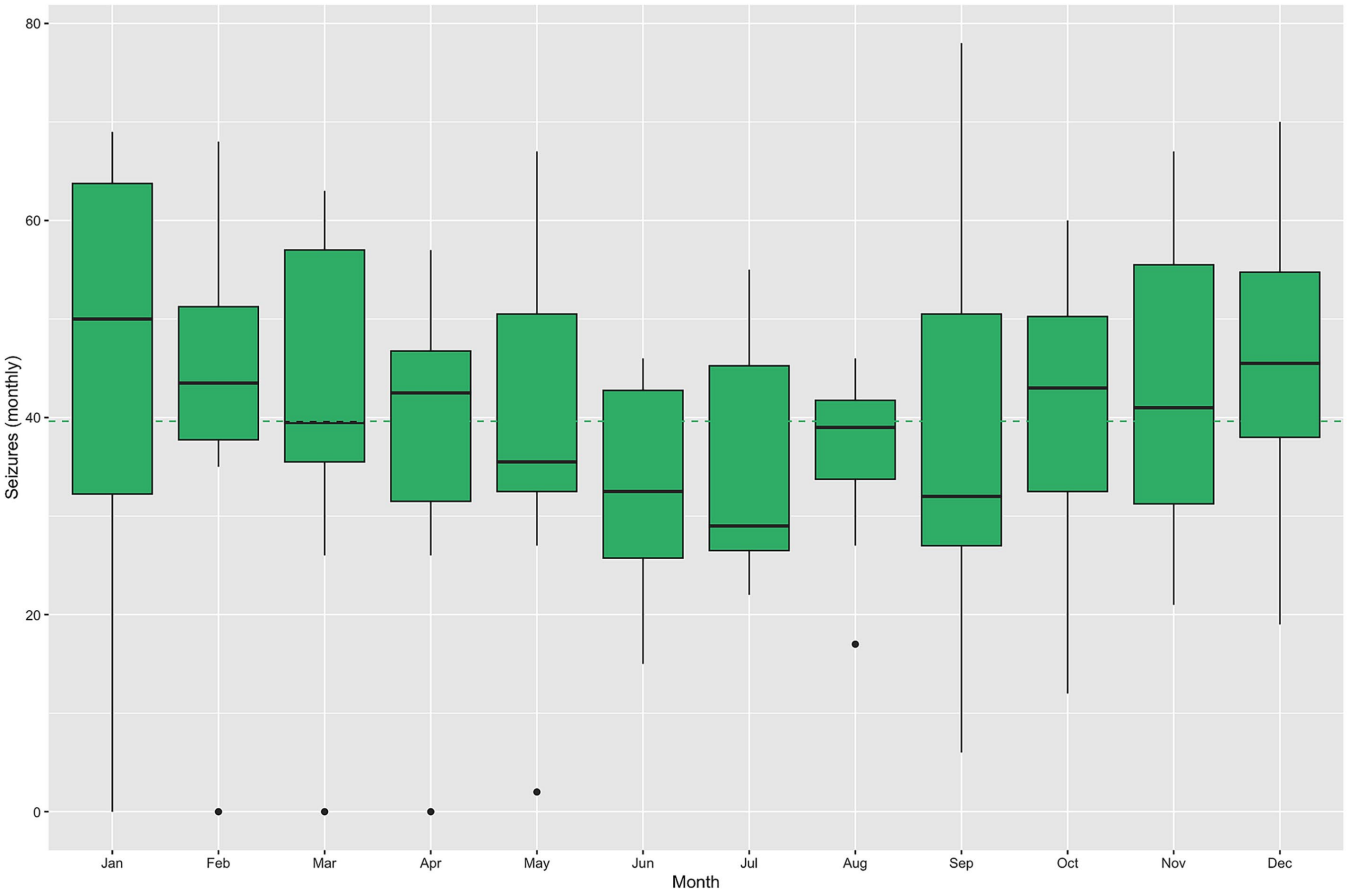
Distribution of daily weather measures and monthly cumulated precipitation by month, 2010–2019. The dashed lines identify the overall mean of each measurement in the color detailed in the corresponding legend.

**Figure 3:**
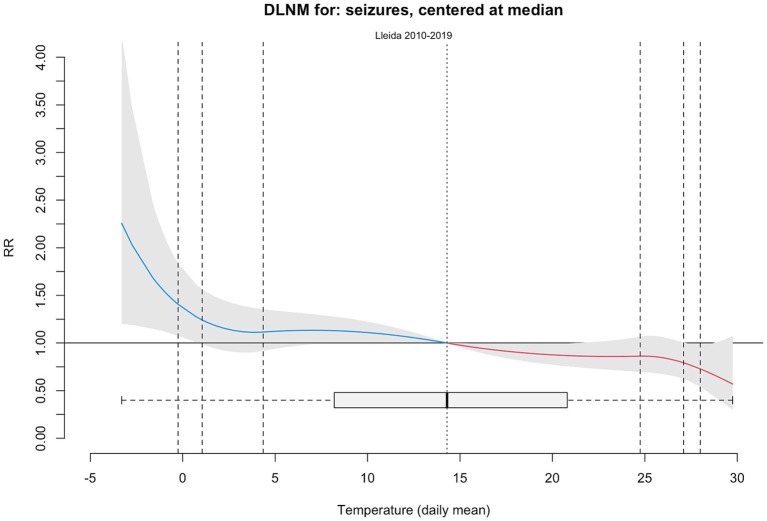
Distribution of daily pollution measures by month, 2010–2019. The dashed lines identify the overall mean of each measurement in the color detailed in the corresponding legend. The red lines identify poor air quality measures as those above red lines.

The monthly distributions of meteorological and air pollution variables are shown in Figures 2, 3, respectively. As illustrated in Figure 1, seizure-related admissions followed a convex seasonal pattern, with peaks in the coldest months—those with lower temperatures and solar irradiation, higher relative humidity and atmospheric pressure, and more days with poor air quality driven by elevated NO_2_ concentrations.

**Figure 4:**
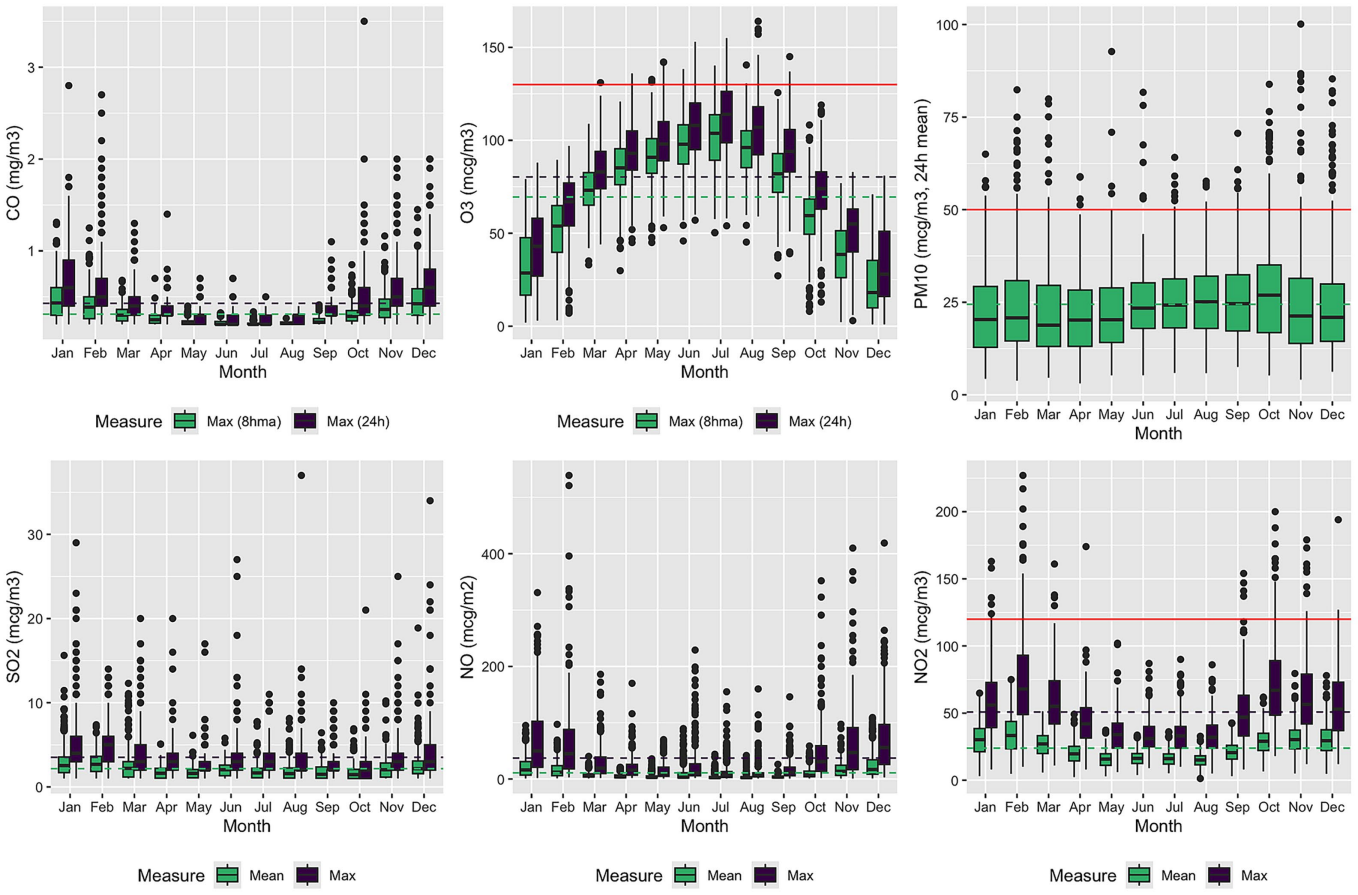
Estimated overall cumulative relationship between seizures and exposure to temperature and NO_2_ concentrations. The three plots show the results of modeling a possible 3-, 7- or 21-days lag delay.

The exposure–response curves (Figure 4) revealed that seizure risk increased significantly on days with extremely low temperatures and high NO_2_ concentrations. Mean daily temperatures below the 2.5th percentile were associated with a marked increase in seizure-related admissions for both 7- and 21-day lags, whereas temperatures above the 97.5th percentile were associated with a decreased risk. Similarly, NO_2_ concentrations above the 99th percentile were associated with an elevated seizure risk. The percent relative effects at 3-, 7-, and 21-day lags are summarized in Table 2.

**Table 2:** Percent change of relative risk associated with weather and pollution exposures.

	Lag	Extreme values (2.5th and 97.5th percentiles)		Extreme values (1st and 99th percentiles)		minY
		Low (P2.5 [95% CI])	High (P97.5 [95% CI])	Low (P1 [95% CI])	High (P99 [95% CI])	
Temperature (daily mean)	3	24.2 [−1.5, 56.5]	−20.8 [−37.6, 0.6]	40.6 [7.6, 83.8]*	−27.2 [−46.1, −1.7]*	P50r
Temperature (daily minimum)	21	52.6 [−8.6, 154.5]	−16.2 [−33.5, 5.5]	107.5 [16.8, 268.8]*	−40.7 [−63.9, −2.8]*	P76
Temperature (daily maximum)	21	58.7 [1.9, 147.2]*	−30.1 [−50.2, −1.9]*	38.8 [−20.1, 141.1]	−29.5 [−55.2, 11]	P50r
Relative humidity (daily mean)	21	−1.2 [−37.4, 55.9]	1.2 [−32.6, 52.1]	28.3 [−24.3, 117.6]	−7 [−43.8, 53.9]	P73
Relative humidity (daily minimum)	21	5.1 [−35.4, 71]	−6.6 [−42.2, 50.8]	28 [−28.2, 128]	−5.7 [−50.5, 79.6]	P50r
Relative humidity (daily maximum)	21	5 [−39.3, 81.4]	−30.5 [−55.5, 8.5]	5.6 [−43.4, 97]	−51.3 [−74.2, −8.1]*	P67
Pressure (daily mean)	21	−8.8 [−42.6, 44.8]	−12.8 [−38.5, 23.7]	−24.2 [−56.9, 33.3]	5.5 [−30.3, 59.5]	P50r
Pressure (daily minimum)	21	−13.4 [−44.3, 34.5]	−15.8 [−40.8, 19.8]	−25.6 [−56.8, 28.2]	8.9 [−29.8, 69]	P50r
Pressure (daily maximum)	21	−9.8 [−43.1, 43.1]	−12.5 [−38, 23.4]	−19.3 [−53, 38.6]	4.6 [−29.9, 56]	P50r
Precipitation	21	0 [0, 0]	−19.2 [−56.9, 51.7]	0 [0, 0]	−44.5 [−78.7, 44.4]	P50r
Solar irradiation	21	95.8 [2.8, 273]*	40.9 [−24.6, 163.2]	97.9 [−7.1, 321.7]	45.6 [−31.5, 209.5]	P61
Wind velocity (daily mean)	3	−4.7 [−22.5, 17.1]	−7.7 [−28, 18.2]	−21.2 [−47, 17.1]	−0.4 [−25.1, 32.4]	P35
Wind velocity (daily maximum)	21	59.5 [−10.2, 183.2]	51.6 [−16.3, 174.8]	93.9 [−23.2, 389.7]	62.4 [−30.7, 280.5]	P30
CO (daily 8 h ma maximum)	21	18.6 [−10.5, 57]	16.7 [−30.1, 94.6]	18.6 [−10.5, 57]	16.8 [−34.1, 107]	P50r
CO (daily maximum)	21	8.3 [−18.1, 43.1]	5.7 [−37.2, 77.8]	8.3 [−18.1, 43.1]	11 [−38.2, 99.1]	P50r
NO_X_ (daily mean)	21	32.2 [−14.4, 104.2]	−27.7 [−55.6, 17.6]	13.6 [−34.6, 97.2]	−19.3 [−56.6, 49.9]	P41
NO_X_ (daily maximum)	7	10.4 [−30.7, 75.8]	−7 [−48.9, 69.4]	−4.3 [−52.9, 94.3]	−5.6 [−53.2, 90.1]	P50r
O_3_ (daily 8 h ma maximum)	21	23.6 [−32.8, 127.5]	−0.7 [−32.1, 45.1]	9.1 [−51.7, 146.3]	−24.7 [−53.7, 22.2]	P63
O_3_ (daily maximum)	7	41 [−5.2, 109.8]	9.3 [−16.3, 42.8]	45.5 [−15.1, 149.3]	−10.3 [−38.8, 31.5]	P67
PM_10_ (daily mean)	21	−21.8 [−50.7, 23.9]	−28.6 [−57.5, 20]	−12.1 [−54.1, 68.1]	−22.8 [−54.8, 31.9]	P50r
SO_2_ (daily mean)	21	−14 [−33, 10.4]	2.3 [−35.5, 62.2]	−14 [−33, 10.4]	5.5 [−43.4, 96.6]	P50r
SO_2_ (daily maximum)	21	−14.8 [−35.5, 12.7]	13.4 [−34.3, 95.6]	−14.8 [−35.5, 12.7]	17.2 [−37.9, 121.4]	P50r
NO (daily mean)	3	3.7 [−19.1, 32.9]	6.2 [−15.7, 33.8]	2.7 [−24.9, 40.4]	7.6 [−17, 39.6]	P45
NO (daily maximum)	21	4.9 [−33.1, 64.7]	−8.6 [−48, 60.7]	4.9 [−33.1, 64.7]	−7.5 [−52.3, 79.5]	P50r
NO_2_ (daily mean)	7	4.5 [−16.9, 31.2]	19.9 [−10.8, 61.2]	−6.5 [−32.2, 28.8]	41.6 [3, 94.6]*	P80
NO_2_ (daily maximum)	7	5.5 [−18.7, 36.9]	28.6 [−9.3, 82.5]	−8.7 [−39.9, 38.9]	39.6 [−3.3, 101.6]	P79

The orange squares represent the significant values in the Low columns, and the green squares represent the significant values in the High columns.

**Figure 5:**
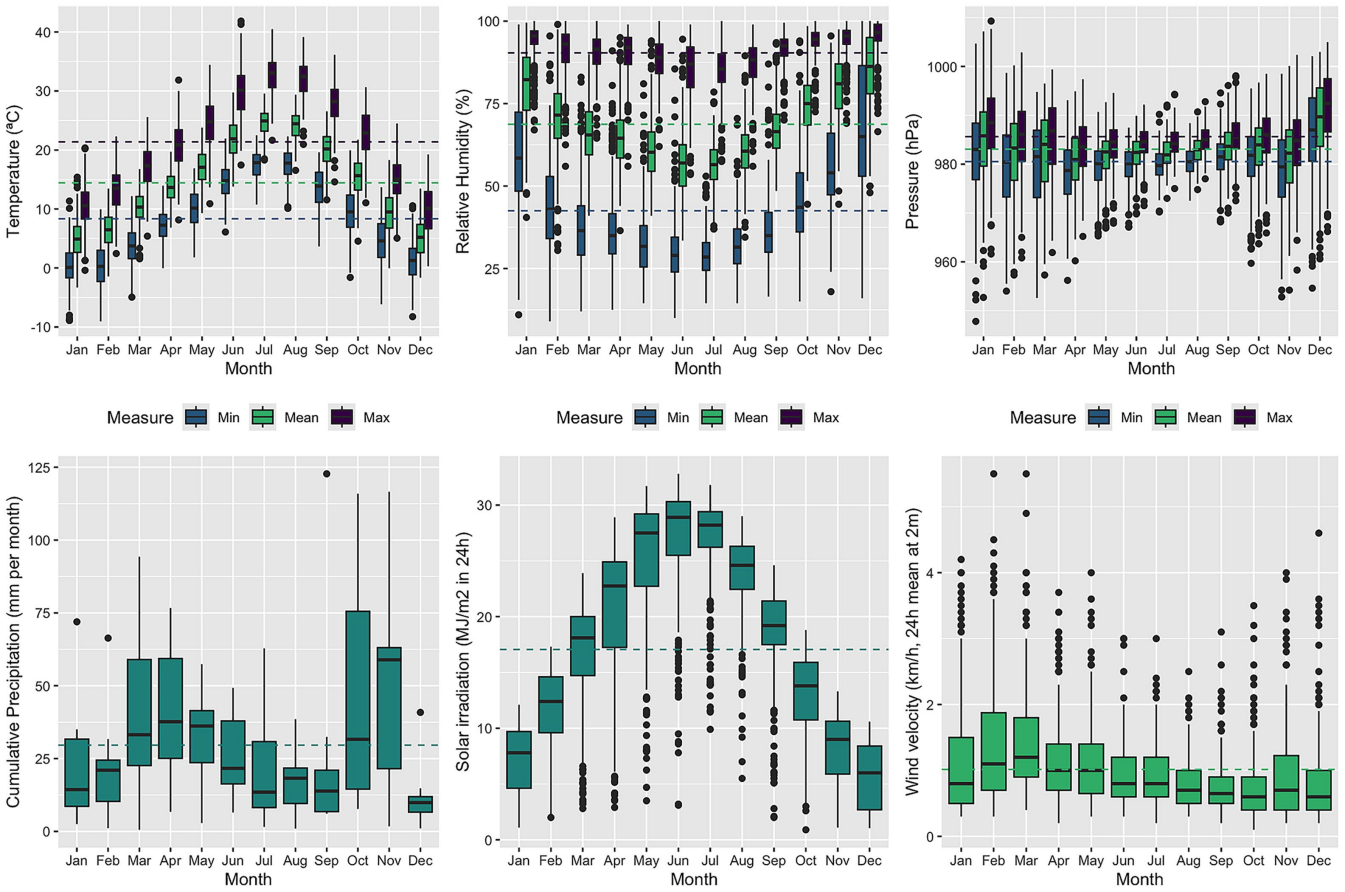
Estimated overall cumulative relationship between seizures and exposure to NO_2_ concentrations adjusted by temperature. The three plots show the results of modeling a possible 3-, 7- or 21-days lag delay.

The association between high daily mean NO_2_ exposure and seizure admissions remained significant after adjusting for temperature using natural spline terms (Figure 5).

## Discussion

In our study, we detected a significant association between the onset of epileptic seizures and both high levels of nitrogen dioxide (NO_2_) and low temperatures. While the primary objective was to explore the relationship between environmental factors and seizure-related emergency visits, the geographic dispersion of hospitals across the health region may have introduced variability in exposure. Although air quality data were obtained from a single monitoring station, seizure-related visits from rural areas—potentially located farther from the monitoring site—were also included. This approach reflects the real-world healthcare utilization patterns of the region but may reduce spatial specificity.

Previous research on meteorological influences on seizure occurrence has produced mixed results. An initial study by Doherty et al. linked seizure frequency to rapid atmospheric pressure changes greater than 5.5 hPa per day (20), though subsequent analyses by the same author did not confirm this association. Other studies have reported associations with high daily temperature fluctuations and sunnier days (21), while high humidity, higher temperatures, and cloudier conditions were suggested to have protective effects (22). In contrast, our findings indicate an increase in emergency consultations for seizures during colder periods.

The greater frequency of seizures during colder months may be related to several physiological and environmental mechanisms. Low temperatures can induce oxidative stress, vascular changes, and sympathetic activation, all of which may reduce the seizure threshold. Cold weather is also associated with higher rates of respiratory infections and fever, which can destabilize seizure control in predisposed individuals. Moreover, reduced physical activity, decreased sunlight exposure, and alterations in circadian rhythm during winter months may further contribute to neuronal excitability.

Regarding NO_2_, higher concentrations during colder seasons can be explained by increased emissions from heating systems and the combustion of fossil fuels, as well as by thermal inversion phenomena, which trap pollutants near the ground and limit atmospheric dispersion. These mechanisms are consistent with studies from other temperate regions showing seasonal peaks of NO_2_ during winter months.

Air pollution remains a major global public health concern. Extensive evidence links it to respiratory (23) and cardiovascular diseases (24), and growing research indicates potential neurological effects (25), including stroke, migraines, cognitive impairment, neurodegenerative disorders, and psychiatric conditions. However, relatively few studies have examined its association with seizures. According to a recent review, six epidemiological studies have evaluated air pollution and seizure risk (26), most of which found that elevated atmospheric pollutant levels (CO, NO_2_, O_3_, SO_2_, PM_10_, and PM_2.5_) may increase hospitalization rates for seizures, particularly with NO_2_ exposure (27).

One study using electroencephalogram (EEG) monitoring reported a positive association between CO exposure and subclinical epileptic activity, although no significant relationship was found for NO_2_, O_3_, SO_2_, or PM_10_ (28). In that Australian study, pollutant concentrations were generally within acceptable limits, contrasting with findings from areas with higher ambient pollution such as China and Chile. In our setting, CO levels remain low, but NO_2_ concentrations frequently exceed recommended thresholds (29).

Ambient particulate matter rarely acts in isolation. In real-world environments, PM_2.5_ and PM_10_ often coexist and interact with gaseous pollutants such as NO_2_ and O_3_, producing complex atmospheric mixtures with synergistic toxic effects. NO_2_ can adhere to the surface of fine particles, enhancing their oxidative potential and enabling reactive nitrogen species to reach deep into the respiratory tract and circulation. Likewise, photochemical reactions involving O_3_ can alter the composition of particulates, generating secondary organic aerosols and reactive oxygen species. Together, these exposures amplify oxidative stress, systemic inflammation, and endothelial dysfunction—mechanisms that not only affect respiratory and cardiovascular systems but may also promote neuroinflammation and lower the seizure threshold.

The biological plausibility of these findings is supported by emerging evidence. Air pollutants such as NO_2_ and PM_2.5_ can trigger oxidative stress, neuroinflammation, and glial activation, disrupting neuronal homeostasis and potentially facilitating epileptic discharges. Recent systematic reviews have confirmed that air pollution, particularly NO_2_, is associated with increased risks of unprovoked seizures and new-onset epilepsy (8). Similarly, a scoping review by Sharma et al. (2) highlighted the contribution of both air quality and temperature to seizure occurrence, reinforcing the relevance of our findings.

Although the retrospective nature of the data is a limitation, large administrative datasets remain valuable for studying low-frequency conditions such as seizures. They allow for robust statistical modeling and population-level inference. The meteorological data were collected from multiple stations within the region, representing local climatic variability. Nevertheless, rural areas—where pollution levels are generally lower—were underrepresented, as only one station provided NO_2_ data. In rural settings, ozone levels tend to be higher, whereas NO_2_ and particulate concentrations are lower. Given population distribution, however, most seizure-related admissions occurred within the urbanized health region, where environmental conditions are similar to those of the provincial capital.

## Limitations

Several limitations should be considered. First, seizure-related admissions were identified using ICD-10-CM codes G40, which capture the majority of epileptic seizures, and R56 codes (febrile convulsions) were excluded. However, some G40-coded cases may correspond to non-epileptic events such as syncope or other transient loss-of-consciousness episodes. Second, individual-level confounders—such as medication adherence, comorbidities, socioeconomic status, and lifestyle factors—could not be included due to the nature of the registry data. Nonetheless, the time-series design inherently controls for stable population characteristics, minimizing the risk of major confounding. Third, exposure was assigned based on fixed outdoor monitoring stations, without accounting for indoor pollution or personal activity patterns, which may have led to exposure misclassification.

## Conclusion

Our findings suggest that both low temperatures and elevated NO_2_ concentrations are independently associated with an increased risk of seizure-related emergency visits. These results support the hypothesis that environmental conditions—particularly during colder months—can influence seizure occurrence through physiological and inflammatory mechanisms. Moreover, the seasonal rise in NO_2_ levels during winter likely amplifies this effect through combined exposure pathways.

This study contributes to the growing body of evidence linking environmental factors to neurological health and highlights the need to integrate air quality and meteorological data into epilepsy surveillance and preventive health strategies. Future research should incorporate individual-level clinical and behavioral data to better clarify causal pathways and identify vulnerable subpopulations. Strengthening public health policies aimed at reducing air pollution could have a meaningful impact not only on cardiovascular and respiratory outcomes but also on neurological diseases such as epilepsy.

## Data Availability

The raw data supporting the conclusions of this article will be made available by the authors, without undue reservation.
